# Targeting Diet and Exercise for Neuroprotection and Neurorecovery in Glaucoma

**DOI:** 10.3390/cells10020295

**Published:** 2021-02-01

**Authors:** James R. Tribble, Flora Hui, Melissa Jöe, Katharina Bell, Vicki Chrysostomou, Jonathan G. Crowston, Pete A. Williams

**Affiliations:** 1 Department of Clinical Neuroscience, Division of Eye and Vision, St. Erik Eye Hospital, Karolinska Institutet, 171 64 Stockholm, Sweden; james.tribble@ki.se (J.R.T.); melissa.joe@ki.se (M.J.); 2 Centre for Eye Research Australia, Royal Victorian Eye and Ear Hospital, Melbourne, VIC 3002, Australia; f.hui@unimelb.edu.au (F.H.); jonathancrowston@duke-nus.edu.sg (J.G.C.); 3 Department of Optometry & Vision Sciences, The University of Melbourne, Melbourne, VIC 3053, Australia; 4 Singapore Eye Research Institute, Singapore National Eye Centre, Singapore 168751, Singapore; katharina.c.bell@seri.com.sg (K.B.); v.chrysostomou@duke-nus.edu.sg (V.C.); 5 Duke-NUS Medical School, Singapore 169857, Singapore

**Keywords:** glaucoma, optic nerve, retinal ganglion cells, diet, exercise, NAD, pyruvate, lactate, recovery, ketogenesis

## Abstract

Glaucoma is a leading cause of blindness worldwide. In glaucoma, a progressive dysfunction and death of retinal ganglion cells occurs, eliminating transfer of visual information to the brain. Currently, the only available therapies target the lowering of intraocular pressure, but many patients continue to lose vision. Emerging pre-clinical and clinical evidence suggests that metabolic deficiencies and defects may play an important role in glaucoma pathophysiology. While pre-clinical studies in animal models have begun to mechanistically uncover these metabolic changes, some existing clinical evidence already points to potential benefits in maintaining metabolic fitness. Modifying diet and exercise can be implemented by patients as an adjunct to intraocular pressure lowering, which may be of therapeutic benefit to retinal ganglion cells in glaucoma.

## 1. Introduction

Glaucoma is the leading cause of irreversible vision loss, affecting an estimated 80 million people worldwide [1]. This neurodegenerative disease leads retinal ganglion cells to undergo a progressive dysfunction and loss, resulting in visual impairment. Up to 40% of patients will go blind in at least one eye [2]. Three leading risk factors include: age, high intraocular pressure (IOP), and genetics. With an increasingly aged population, glaucoma prevalence is set to increase. IOP is thus far the only treatable risk factor for glaucoma, and current management strategies all depend on lowering IOP. Unfortunately, many patients are refractory to IOP-lowering therapies, and we currently have no additional ways to manage disease progression. Normal tension glaucoma (NTG) patients display a similar retinal ganglion cell loss in the absence of high IOP, yet rely on the same IOP-lowering approaches as the only available strategy. Glaucoma is a complex, polygenic disease with large environmental interplay, with only ~5% of primary open-angle glaucoma (POAG, associated with high IOP) cases being monogenic [3]. Future corrective gene therapies for glaucoma will therefore not address the needs of the majority of glaucoma patients. Population studies and gene-association studies have identified an increased risk of glaucoma subtypes associated with ethnicity, and a number of genes that largely impact ocular anatomy, IOP control, trabecular meshwork, and metabolism (~30 genes associated with POAG; ~10 with primary closed angle glaucoma (PACG) [4]). Genes such as *ABCA1* and *PMM2* (involved in carbohydrate and lipid metabolism and anabolism), are known to be expressed in the retina and optic nerve [5], and other metabolic gene variants (e.g., *ELOVL5*; fatty acid metabolism) have been identified [6]. Pathway analyses of gene variations have implicated lipid metabolism and mitochondrial metabolism in POAG [5,7]. In animal models of glaucoma, a growing body of evidence supports early metabolic disruption to retinal ganglion cells and their supportive networks [8,9,10,11,12], and a number of metabolic interventions have demonstrated robust neuroprotective effects [8,12,13,14]. While many of these remain at the pre-clinical stage or are in early clinical testing, they offer a potential route to combat glaucoma progression. Circumvention of metabolic decline may offer a viable therapeutic strategy to manage glaucoma progression in combination with existing IOP-lowering tools. Such interventions include exercise, alternative energy sources such as lactate/pyruvate, increasing metabolic cofactors such as NAD, modifying lipid metabolism through ketogenesis, and controlling dietary influencers that may impact metabolism or IOP. In this review, we summarize these metabolism-modulating approaches, with a focus on human studies conducted in the last 15 years (including relevant pre-clinical experiments), and comment on their applicability for neuroprotection and neurorecovery in glaucoma. We focus on the neuronal outcomes, but many of these interventions likely also modify other aspects of glaucoma pathophysiology, and systemic effects that may influence the eye. These include the regulation of vasculature tone [15], oxygen demand [16], and inflammation [17], which will not be reviewed here, but are likely to be important in the context of glaucoma and glaucoma management. Neuroinflammation is an early and sustained component of glaucoma [17], as are vascular compromise and hypoxia [9,15,18,19]. The modulation of neuroinflammation in neurodegenerative disease through exercise has been reviewed in detail elsewhere [20,21]. Hypoxia is known to drive adaptive metabolic changes to cope with reduced oxygen availability (see [22] for a comprehensive collection), which dietary supplementation could positively impact by providing alternative energy sources (e.g., fuel for anaerobic glycolysis), thereby limiting the impact of these metabolic changes. For the purpose of retinal ganglion cell neuroprotection in glaucoma, diet and exercise represent achievable lifestyle changes that could be implemented to some degree by most patients as suits their circumstances, in addition to ongoing IOP-lowering therapeutics.

## 2. Targeting Exercise for Neuroprotection in Glaucoma

The first association between physical activity levels and glaucoma susceptibility was published in the early 1980s, in which Ivan Goldberg and colleagues reported that individuals with the most robust optic nerves (those with ocular hypertension and no glaucoma) habitually performed substantially higher rates of exercise compared to those with the most vulnerable nerves (those with glaucoma damage in the presence of normal IOP). These data suggested that exercise might modulate the susceptibility of the optic nerve to glaucomatous damage [23]. More recently, a large cohort study of over 27,000 male runners from running clubs across the USA reported a strong dose-dependent association between self-reported incident glaucoma and weekly running habits. Those that ran further distances and ran faster times over 10 km reported less incidences of glaucoma [24]. A major limitation of the study was that glaucoma incidence was self-reported, but this was offset by an impressive cohort size together with the demonstration of dose-dependent outcomes. In a separate prospective population-based study of men and women aged 39 to 79 years, Yip and colleagues found that increased levels of physical activity had a long-term beneficial impact on ocular perfusion pressures, a factor that may play a role in glaucoma risk [25]. However, a causal relationship between ocular perfusion and glaucoma risk has recently been brought into question [26]. The interpretation of such studies is potentially complicated by the fact that those who exercise may have better vision than individuals who are less likely to participate in physical activity. Glaucoma patients routinely perform less exercise than age-matched controls [27].

Elevated IOP remains an important risk factor in glaucoma. A number of studies have shown that exercise transiently lowers IOP. More recent published evidence suggests that exercise may also lower baseline IOP for a prolonged period of time [28]. The influence of dynamic exercise on IOP remains contentious, as increased aqueous production due to hydration before and during exercise sessions, as well as elevated blood pressure, may all exacerbate mechanisms that result in IOP elevation.

Experimental studies have the capacity to provide more direct evidence that exercise can protect against various forms of retinal ganglion cell and optic nerve injury. In aged mice, forced exercise by swimming significantly improved retinal ganglion cell functional recovery after an acute pressure elevation, even when initiated 24 h after injury [29]. Improved retinal ganglion cell function in exercised mice was associated with preservation of synaptic connections within the inner plexiform layer, reduced loss of retinal ganglion cell somas, and reduced astrocytic gliosis and microglial activation [30]. In a different animal injury model, rats that performed treadmill exercise before and after optic-nerve transection showed significantly increased retinal ganglion cell survival [31]. The protective effects of exercise in these experimental systems were subsequently found to be critically dependent on brain-derived neurotrophic factor (BDNF) signaling. Whereas BDNF levels were significantly reduced in the injured retinas of non-exercised mice and rats, BDNF levels were maintained in the injured retinas of exercised animals. Blocking BNDF signaling during exercise by intraperitoneal injections of ANA-12, a BDNF tropomyosin receptor kinase (TrkB) antagonist, abolished the protection of retinal ganglion cells afforded by exercise [30,31]. Furthermore, mice carrying a heterozygous deletion of the BDNF gene were not protected by exercise. Other neurotrophins, including neurotrophic growth factor (NGF) and insulin-like growth factor-1 (IGF-1), have demonstrated neuroprotective effects on retinal ganglion cells [32,33]. However, their roles in exercise-mediated protection are less established. *Ngf* expression following exercise appears to differ according to brain region; increased expression has been identified as an early, transient response in some cases, and only after longer-term exercise in others [34,35,36]. NGF does not appear to be elevated to the same degree as BDNF in response to exercise [37]. IGF-1 is produced in the liver and muscles, and can permeate the blood-brain barrier; exercise increases circulating IGF-1 levels and increases its uptake to the brain in rats [38,39,40]. Although these additional neurotrophins are attractive potential future treatments for glaucoma in the form of drug or gene therapies, they are still to be definitively clinically tested, and their role in existing glaucoma patients assessed. Even though exercise capacity may be reduced in individuals with vision impairment, there is a need to further investigate these associations with a view to understanding the underlying molecular pathways and highlight potential mimetics that could mediate similar levels of protection without the patient necessarily needing to get off the couch.

## 3. Targeting Diet for Neuroprotection in Glaucoma

### 3.1. NAD

Nicotinamide adenine dinucleotide (NAD) is an essential cellular metabolic factor. NAD acts as a cofactor in several REDOX reactions, and functions as a substrate for several protein classes, including NAD consumers: poly (adenosine diphosphate ribose) polymerases (PARP; involved in DNA damage repair [41]), sirtuins (SIRT; involved in transcription regulation, energy metabolism modulation, and DNA repair [42]), and sterile alpha and Toll/interleukin-1 receptor motif-containing protein 1 (SARM1; a regulator in neurodegenerative processes [43]). NAD levels decline during normal aging [44], and a disruption of NAD homeostasis is associated with several age-related neurodegenerative diseases, such as glaucoma, Alzheimer’s disease, and Parkinson’s disease [45,46].

There are three known synthesis pathways for NAD in mammalian systems: the de novo pathway (also called the kynurenine pathway), the Preiss–Handler pathway, and the salvage pathway. The de novo pathway synthesizes tryptophan to NAD through an eight-step process. The Preiss–Handler pathway uses nicotinic acid and the enzyme nicotinic acid phosphoribosyltransferase (NAPRT) to synthesize nicotinic acid mononucleotide (NaMN), which is converted to nicotinic acid adenosine diphosphate (NaAD) through nicotinamide mononucleotide adenylyltransferases (NMNATs), and finally converted to NAD through NAD synthase (NADS). In neurons, NAD synthesis occurs predominantly through the salvage pathway by recycling nicotinamide (the amide version of nicotinic acid). In the salvage pathway, nicotinamide is converted to nicotinamide mononucleotide (NMN) by nicotinamide phosphoribosyltransferase (NAMPT), which is then converted to NAD by NMNATs. There are three isozymes of NMNATs with different subcellular localization and tissue abundances; NMNAT1 is ubiquitous and localized to the nucleus, NMNAT2 is localized to the Golgi body and cytosolic compartments and is expressed exclusively in neurons and hair follicles, and NMNAT3 is localized to the mitochondria [47] (although whether this occurs in mature in vivo mammalian systems is debatable). NAD homeostasis and NMNAT expression have a clear role in neuronal survival, axon degeneration, and neurodegenerative diseases in both animal models and humans [48,49,50,51,52,53]. NAD consumers, such as PARPs, SIRTs, and SARM1, may also play an important role in glaucoma [54,55], particularly as NAD homeostasis is altered, which may disrupt the wider roles of metabolic regulation that these proteins play. For example, SIRTs modulate electron transport chain proteins, mitochondrial biogenesis, and mitochondrial lipid metabolism [56]. SIRT1 activation delays retinal ganglion cell loss from optic nerve crush and from hypoxic stress [57,58], whereas SARM1 deficiency protects retinal ganglion cell integrity, but does prevent cell death in optic nerve crush [59].

In human studies, lower niacin intake is associated with NTG [60] (niacin broadly refers to nicotinic acid, nicotinamide, and derivatives of nicotinamide), and lower concentrations of nicotinamide have been measured in the sera of POAG patients [61]. This suggest that dietary counseling or supplementation of NAD precursors could be a useful intervention for glaucoma patients. Several precursors, such as nicotinic acid, nicotinamide riboside, NMN, and nicotinamide have undergone clinical testing for safety assessment and in various pathologies [62,63], of which nicotinic acid demonstrates the worst adverse effects (it induces flushing within 30 min after oral administration [63,64,65]). NAM is the only precursor that has been tested in the context of glaucoma, for which we have demonstrated improved inner retinal function of existing primary glaucoma patients who were administered up to 3 g/day [66]. In preclinical experiments in the DBA/2J mouse model of glaucoma (which develops an age-related, ocular hypertensive, pigmentary glaucoma resulting from iris pigment dispersion), we demonstrated an age- and IOP-dependent decline in NAD in the retina, dysregulation of key metabolic pathways, and loss of internal mitochondrial structure and overall size. Supplementation with nicotinamide (equivalent to ~2.5 g/day in a 60 kg human) prevented these disease features, as well as loss of retinal ganglion cells. Further studies have recapitulated this retinal ganglion cell neuroprotection [12,67] and protection of visual function [68], and demonstrated that nicotinamide is rapidly taken up by retinal neurons and mobilized to maintain supra-physiological levels of NAD in retinal ganglion cell relevant tissues [67]. Crucially, nicotinamide supplementation of non-glaucomatous animals did not significantly alter metabolic profiles [67], which is in line with nicotinamide’s excellent clinical safety profile and long history of use in other contexts. However, more studies are required to determine the effects of long-term administration on the progression of glaucoma.

The understanding of how NAD and its precursors are metabolized and absorbed in the gastrointestinal tract and distributed in the body will be crucial to titrate effective doses. New evidence shows that orally administrated amidated precursors (nicotinamide riboside, nicotinamide, and NMN) are deaminated by the microbiota in the gastrointestinal tract prior to absorption [69]. Additionally, treating mice with isotope-labeled NMN increased unlabeled NAD, which suggests that there is an indirect impact of NMN on NAD levels, rather than full conversion [70]. Circulating nicotinamide, rather than other NAD precursors, appears to maintain systematic NAD homeostasis, with other precursors synthesized to nicotinamide in the liver [71]. Supporting this, NMN and nicotinamide riboside fail to cross the blood–brain barrier [71], and as such, unlike nicotinamide, lack accessibility to CNS tissues. Further investigation is required to fully understand the metabolism and distribution of NAD precursors, but an increase in nicotinamide seems most likely, given current understanding. Direct supplementation with nicotinamide therefore offers the simplest route to increasing NAD in the retina.

### 3.2. Lactate/Pyruvate

Both pyruvate and lactate are glycolysis end products and serve as important energy sources for many cells and tissues, including neurons. Pyruvate is still considered the main product generated from the breakdown of glucose by glycolysis, and can either be converted to acetyl-CoA (to enter the Krebs cycle) or alternatively be converted to lactate. Whereas pyruvate has long been recognized as a beneficial glycolysis-derived product, lactate production was considered a waste product of glycolysis as a consequence of oxygen deficiency in exercising muscle cells. This paradigm, however, is long outdated, with numerous studies demonstrating the production of the L-enantiomer of the lactate anion within aerobic conditions. In mammals, including humans, L-lactate serves as an important energy source in the form of a substrate for gluconeogenesis, and can act as a signaling molecule with autocrine-, paracrine-, and endocrine-like effects. Lactate is utilized as an energy source throughout the whole body by means of a global lactate shuttle. Intracellularly, glycolysis can be linked to the mitochondrial respiration system through an intracellular lactate shuttle. Different forms of these lactate shuttles have recently been reviewed by G. Brooks [72].

Lactate has been recognized as an important energy source for neurons in the CNS. The monocarboxylate transporter 1 (MCT1) allows transport of lactate through the blood–brain barrier, as well as the inner and outer blood–retina barrier [73] in a concentration-dependent manner [74]. There is ongoing debate as to whether retinal ganglion cells preferably utilize lactate or glucose as their primary energy source. In vitro studies show that retinal ganglion cells can utilize either glucose, lactate, or pyruvate as energy sources [16,75]. MPC1 and MPC2 are present at high levels in retinal ganglion cells across a number of species, suggesting an importance of pyruvate metabolism [12]. In vascularized retina, photoreceptors express high levels of lactatedehydrogenese A (which converts pyruvate into lactate), indicating the capacity for photoreceptors to convert pyruvate to lactate. This could provide photoreceptors with energy [76], but also permit photoreceptors to serve as valuable lactate providers for other retinal cells [16]. Müller cells could play an important role in shuttling lactate from photoreceptors to the inner retinal areas, either directly, which is supported by the presence of different MCT receptors on Müller cells [77], or as glucose, from the breakdown of glycogen stores [78]. However, lactate also appears to be a preferred energy source for Müller cells, and increases their function, enhancing their survival when supplied exogenously [79]. Müller cell metabolism of lactate may provide an important source of redundancy against declining retinal ganglion cell metabolism [80]. Astrocytes in the inner vascularized retina could also provide lactate to retinal ganglion cells, using an astrocyte neuron-lactate shuttle similar to that which has been described in the brain [81]. Astrocytes wrap around inner retinal vessels, allowing them to take up glucose from the blood [78]. Direct evidence for such a shuttle mechanism in the retina remains outstanding, but is evident in optic nerve axons in which oligodendrocytes provide lactate as fuel [82]. Lactate serves as an energy supply for the brain not only during exercise, but also during rest. Arterial blood levels of lactate are below 1 mmol/L at rest, but increase up to ~8 mmol/L during moderate exercise [83] and up to 30 mmol/L during extended exercise [84], thereby significantly increasing the blood lactate:pyruvate ratio. An elevated lactate:pyruvate ratio can increase CNS blood flow, and thus increase availability of this energy source in neuronal tissues.

A number of studies have demonstrated a neuroprotective role for pyruvate in experimental models of neurodegeneration, including Parkinson’s disease and ischemic brain injury [85,86]. Pyruvate has been shown to also reduce oxidative stress in an ex vivo cultured retina model [87]. Neuroprotective effects of lactate and pyruvate on retinal ganglion cells have been described in cell-culture models [12,88], including under glucose deprivation. Analysis of optic nerves from glaucomatous DBA/2J mice and from a bead-induced ocular hypertension model in the mouse showed reduced levels of L-lactate, a reduction in MCT2 receptor protein levels, and an increase in the phosphorylated AMPK:AMPK ratio [14]. MCT1 is more abundantly expressed in glial cells, suggesting an export-relevant configuration, and MCT2 receptors are present in neurons (including retinal ganglion cells) showing an import-relevant configuration [89,90]. MCT2 receptor overexpression was demonstrated to provide neuroprotection for retinal ganglion cells in DBA/2J and bead-induced ocular hypertension [90]. Pharmacological inhibition of MCTs blocks the neuroprotective effects of pyruvate on retinal ganglion cells in culture [12]. Recently, we demonstrated a decline in retinal pyruvate with increased exposure to IOP in the DBA/2J, with transcriptional dysregulation of glycolysis and pyruvate metabolism [12]. Long-term oral supplementation with pyruvate significantly reduced optic-nerve degeneration and axon-transport disruption, protected against IOP induced metabolic changes, and enhanced retinal ganglion cell visual function. Retinal ganglion cell death was also significantly reduced in a laser photocoagulation model of sub-acute ocular hypertension [12]. Combining treatment of low-dose nicotinamide and pyruvate increased retinal ganglion cell axon survival further [12]. Synergistic effects of improving glycolytic capacity, where both nicotinamide and pyruvate are important for its regulation, may underlie the protection observed, and indicate the potential for combination therapies. Clinical studies examining the translation of pyruvate as a neuroprotective treatment for glaucoma will be necessary, with one such study currently underway (NCT03797469).

### 3.3. Ketogenesis

A ketogenic diet is high in fat and low in carbohydrates. Understanding the metabolic consequences of this diet has continued to be driven by the epilepsy field, following the observation that fasting reduced epileptic seizures, with these effects abolished by carbohydrate but not fat intake. Under fasting conditions, ketone bodies are produced, and a diet high in fat and low in carbohydrates can mimic these effects (termed a ketogenic diet [91]). A ketogenic diet increases fatty-acid oxidation and gluconeogenesis, and severely limits glycolysis. This results in both an increase in acetyl-CoA (from fatty-acid oxidation) and a diversion of oxaloacetate out of mitochondria to support gluconeogenesis, creating an excess of acetyl-CoA relative to oxaloacetate. Since acetyl-CoA reacts with oxaloacetate in the TCA cycle, acetyl-CoA begin to combine to form acetoacetyl-CoA, and further to HMG-CoA in a non-reversible reaction initiating ketogenesis. HMG-CoA breaks down into acetoacetate (ACA), which can be further reduced to β-hydroxybutyrate (BHB) or decarboxylated to acetone; collectively known as ketone bodies [92]. This predominantly occurs in the liver, from which ketone bodies enter the circulation and cross the blood–brain barrier, but astrocytes also appear to produce ketone bodies utilizing the same mechanism [93,94,95,96]. Ketone bodies and fatty-acid oxidation provide an alternative energy source to glucose and feed into the TCA cycle to continue metabolic intermediate replenishment.

Aside from its use in the management of epilepsy, the ketogenic diet or variants that increase circulating ketone bodies have demonstrated improved outcomes in a number of neurodegenerative conditions and animal models, including Alzheimer’s disease [97,98,99,100] and Parkinson’s disease [101,102,103] (for a more comprehensive review, see [104]). Proposed neuroprotective mechanisms include reduced production of reactive oxygen species, increased mitochondrial biogenesis, bolstered energy reserves (including increasing ATP and phosphocreatine), altered expression of key metabolic enzymes, and disruption of key metabolic processes (including the mammalian target of the rapamycin (mTOR) pathway) [105,106,107,108,109,110]. These effects suggest a protective role for the ketogenic diet in glaucoma, where reactive oxygen species induced damage, mitochondrial loss and metabolic disruption are key and early disease features.

In DBA2/J mice, a ketogenic diet provided significant neuroprotection [14]. At nine months of age (pre-neuronal loss), DBA2/J mice were fed a ketogenic (10.4% protein, 0.1% carbohydrate, and 89.5% fat) or control diet (10.4% protein, 78.2% carbohydrates, and 11.5% fat, to achieve a similar caloric intake). DBA2/J mice on the ketogenic diet demonstrated significantly improved retinal ganglion cell soma and axon survival, axonal transport, compound action potential firing frequency, and reduced astrocyte gliosis over DBA2/J mice on a control diet [14]. The authors identified a number of possible sources for this protection, including upregulated antioxidant responses, improved mitochondrial integrity and up-regulation of mitochondrial biogenesis, increased BDNF, and metabolic rescue. Consistent with a metabolic shift, the optic nerves of DBA2/J mice on the ketogenic diet demonstrated increased L-lactate, creatine kinase, an increase in NAD and the NAD:NADH ratio, and evidence of increased catabolism and downstream activation of the mTOR pathway. Protein expression of MCT1 and MCT2 was increased in DBA2/J mice on the ketogenic diet, suggesting a greater intake of substrate [14]. Whether these are the direct result of ketogenic metabolism or secondary to a retinal ganglion cell (and astrocyte) preservation through mitigating energy crisis remains to be determined; however, these results provide compelling evidence for a neuroprotective effect of a ketogenic diet in experimental glaucoma.

Daily intraperitoneal delivery of lithium ACA and sodium BHB provided no meaningful neuroprotection in a rat model of N-methyl-D-aspartate (NMDA)-induced retinal ganglion cell death. Prophylactic treatment for two weeks and continuing for one week following NMDA injection resulted in only 3–4% increased retinal ganglion cell survival [111]. Since these animals were maintained on standard chow ad libitum, limited conclusions can be drawn from these results regarding a ketogenic diet. Direct application of ketone bodies or a ketogenic diet still produce positive outcomes in many conditions; however, the extent to which circulating ketone bodies in the absence of glucose deprivation exhibit retinal ganglion cell neuroprotective effect remains to be explored. Of note, intravenous infusion of glucose rapidly reverses seizure suppression by a ketogenic diet in epileptic patients [112]. Further experiments will be necessary to determine whether providing ketone bodies either as an adjunct or as a replacement to traditional energy sources elicits neuroprotective effects.

Exploration of the effects of a ketone diet in human patients remains difficult, since the diet has notorious adherence issues. Many patients cannot tolerate the gastrointestinal side effects, nor the palatability, of such a high-fat diet. Moreover, it is difficult to control for and separate the effects of caloric intake, particularly since it affects a number of appetite-controlling mechanisms [113,114]. A modified Atkins diet (where carbohydrates remain low, but fat content is reduced and protein content increased relative to the ketogenic diet) has proved efficacious in controlling epilepsy, with a higher adherence [115,116,117]. A recent evaluation of a low-carbohydrate, high-fat and -protein diet in POAG demonstrated some association with reduced visual field loss. In this study, data were taken from the Nurse’s Health Study, the Health Professionals Follow-Up Study, and the Nurse’s Health Study II, in which the participants’ daily intake by nutrient was estimated from validated food frequency questionnaires. Participants were scored and divided based on adherence to a low-carbohydrate diet, and patients were stratified based on total carbohydrate, total fat, and total protein intakes, and further separated based on the source of dietary fat and protein (animal or vegetable). There was no overall association between adherence to a low-carbohydrate diet and POAG risk. There was an association with paracentral visual field loss in a low-carbohydrate, high-fat and -protein diet from vegetable sources, representing a ~20% reduced risk of this POAG subtype. The authors suggest that this supports findings of mitochondrial and lipid metabolism dysfunction in this POAG subtype, NTG, and Leber’s hereditary optic neuropathy [5,6,7,118]. Clinical trials employing controlled ketogenic or derivative diets will be necessary to fully assess their effects on glaucoma progression and evaluate their role as effective therapeutic strategies. Nevertheless, these works add further evidence to the hypothesis of metabolic disruption as a key component of glaucoma pathophysiology.

### 3.4. Other Common and Manageable Dietary Influencers

#### 3.4.1. Caffeine

Caffeine is the most widely consumed psychoactive stimulant and is produced naturally by at least 30 different plant species, with high levels in coffee (*Coffea arabica* and *Coffea canephora*) and tea (*Camellia sinensis*), as well as low levels in chocolate (*Theobroma cacao*). Reduction in IOP is the only currently clinically available strategy demonstrated to reduce the risk of glaucoma [119]. Coffee, a very rich source of caffeine in most preparations, elevates IOP transiently following ingestion in different glaucoma population subgroups (~2 mmHg over 2 h), as well as in normotensive healthy patients to a lesser degree [120,121,122,123]. This is likely due to its effects as a phosphodiesterase inhibitor, stimulating aqueous humor production. Although reported coffee consumption correlates with a higher IOP in POAG patients (e.g., Blue Mountains Eye Study [124]), there does not seem to be a significant association in larger studies [125,126]. It is important to note that different populations have different risks to glaucoma and that caffeine can increase IOP to different degrees, and thus it is important to take the individual into account when considering caffeine load and IOP and glaucoma risk. Supporting this, a positive association has been demonstrated between coffee consumption and the development of pseudoexfoliative glaucoma in a large study (>3 cups/day) [127], as well as an association with a higher risk of open-angle glaucoma in a large Korean study (with a higher risk associated in male coffee drinkers [128]).

Tea is another caffeine-rich beverage consumed globally with numerous reported health and energy expenditure benefits. These benefits are likely derived from tea’s complex mix of polyphenols/flavonoids, caffeine, vitamins, and minerals, although the exact mechanisms of its protective effects are yet to be defined [129,130]. The flavonoid content of tea (a major polyphenol class) has been associated with better outcomes in glaucoma [126,131]; which can be potentially attributed to their role in IOP regulation, vascular regulation, or as an antioxidant mechanism [132]. More research is required to identify the role of tea consumption in glaucoma risk, as well as the mechanism of any neuroprotection. As the catechin class of flavonoids are rich in green tea and have known neuroprotective properties (as antioxidants, anti-inflammatory agents, and iron chelators [133,134]) there are a spectrum of possibilities for green tea components that may have beneficial neuroprotective effects in glaucoma.

#### 3.4.2. Alcohol

Alcohol (ethanol) consumption has been demonstrated to acutely lower IOP (e.g., ~3–4 mmHg transient decrease for ~60 min [135]). This likely occurs through multiple secondary mechanisms, including vasopressin suppression (leading to reduced water movement) and hyperosmolarity changes [136]. Acetaldehyde, as a byproduct of ethanol consumption, has been demonstrated to decrease blood-vessel resistance and increase blood flow in the optic nerve head, and this might contribute to a protective effect as well [137]. Larger association studies have failed to find a significant association between glaucoma and alcohol intake, except for the Framingham Eye Study, which did demonstrate a positive association between high alcohol consumption and glaucoma [138,139,140,141]. High alcohol consumption is also associated with other comorbidities for glaucoma (such as diabetes and obesity) that may contribute to negative glaucoma outcomes in this way.

#### 3.4.3. Antioxidants

Additional nutritional factors are likely of great importance for systemic health, as well as retinal and optic nerve health, in glaucoma. The role of different antioxidants (e.g., vitamins A, C, and E; omega 3 and 6; co-enzyme Q_10_; resveratrol; and green tea extract) have been well explored in animal models and are reviewed in detailed elsewhere, with a general trend for mild to moderate neuroprotective effects in isolation and in combination formulations [142]. A number of clinical trials and association studies have been performed to assess antioxidant capacity/dietary intake or antioxidant treatment in glaucoma [143,144,145,146,147,148,149,150,151]. The Osteoporosis Fracture Cohort associated a reduced risk in open-angle glaucoma in women with frequent (>1/month) green collards/kale, carrot, or canned peach consumption [152]. These specific fruit and vegetables are rich in vitamins A, B_2_, and C, which may contribute to this effect. An increase in vitamin B_2_ itself correlated with lower odds of having glaucoma in this study [152], warranting additional studies that examine individual vitamin supplementation in an isolated fashion. Vitamin B_12_ (important for DNA synthesis and fatty-acid and amino-acid metabolism) decreases with age (~10–15% of people >60 years of age are diagnosed as deficient [153]), and has been demonstrated to be low in the sera of POAG patients [154]. These low levels of vitamin B_12_ are highly correlated with higher homocysteine levels (a known oxidant that drives retinal ganglion cell apoptosis in animal models [155]); and hyperhomocysteinemia has been reported in pseudoexfoliative glaucoma patients [154]. Supporting this, long-term methylcobalamin (a vitamin B_12_ homologue) delayed visual field loss in a small cohort of Japanese NTG patients [156].

*Ginkgo biloba* is another commonly taken supplement with reported beneficial effects for glaucoma patients (as well as in other neurodegenerative diseases and general aging [157]). *Ginkgo biloba* exercises a neuroprotective effect in cell and animal models through its capacity as an antioxidant, vasoregulatory effects, and inhibition of nitric oxide activity [158]. Supporting this, a number of smaller studies have demonstrated a beneficial effect of *ginkgo biloba* extract on the visual field, and best corrected visual acuity in open-angle low-pressure glaucoma patients [159,160].

Fatty acids (especially omega-3) play essential roles in cellular metabolism and antioxidant capacity (with omega-3 supplementation increasing systemic antioxidant capacity in humans [161]). Numerous studies have assessed omega-3 and omega-6 intake in large population studies, demonstrating different levels risk associated with their intake [162,163]. The Nurses’ Health Study and the Health Professionals Follow-up Study demonstrated an increased risk of POAG with high omega-3 to omega-6 intake [164], whereas different studies have failed to replicate this effect in context of randomized controlled trials of POAG patients and omega-3 supplementation [165,166]. Again, it may be important to consider the wide effects of these antioxidants, their dosing and bioavailability, and the needs or diagnoses of the individual patient.

#### 3.4.4. Obesity

Obesity is defined by the World Health Organization as a body mass index (BMI) of ≥30 kg/m^2^. The prevalence of obesity and the presence of an obesogenic diet and lifestyle are increasing globally. This is resulting in a significant health and socio-economic burden based on common systemic diseases with obesity as a common risk factor (e.g., diabetes, hypertension, stroke, sleep apnea), as well as more general disability and lack of exercise, which further compounds these negative effects [167]. A high BMI correlates with decreased choroidal perfusion, lower ocular blood flow, increased orbital fat, and elevated IOP in different population studies [168,169,170,171]—all factors that might contribute negatively to glaucoma pathogenesis. Obesity also drives oxidative stress in a more systemic fashion that is likely to negatively impact trabecular meshwork, retinal, and optic nerve health. However, these effects are not conclusive, and inconsistent findings have been demonstrated in different population groups, with the Gangnam Eye Study identifying high BMI as a significant risk factor for POAG [172], and the 2008–2010 Korea National Health and Nutrition Examination Survey identifying an association between high BMI and high IOP [173]. No association was found in the Nurses’ Health Study, the Health Professionals Follow-up Study, the Rotterdam Study, or the Barbados Study [174,175,176]. Again, care should be taken when overgeneralizing these results, considering the difference in the study populations, ethic and genetic backgrounds, and study designs.

## 4. Functional Recovery in Glaucoma

### 4.1. Is It too Late to Change?

Can any of these dietary and exercise interventions improve vision, or is it too late? It is traditionally believed that vision loss in glaucoma is irreversible; however, recent clinical and pre-clinical evidence suggests that retinal ganglion cells have the capacity to recover function [177,178,179]. This has led to the concept of the “injured” retinal ganglion cell, a critical state of plasticity prior to irreversible cell death when retinal ganglion cell dysfunction is potentially reversible [180,181]. Porciatti and Venturi hypothesized that when exposed to chronic stress, retinal ganglion cells have a stress-dependent adaptive response to sustain function and prolong survival. When the cumulative stress (i.e., from elevated IOP, hypoxia, or bioenergetic deficiency) exceeds autoregulatory capacity, retinal ganglion cells become dysfunctional, relative to the amount of stress. By modifying these stressors, it may be possible to improve retinal ganglion cell function and prevent further vision loss.

Retinal ganglion cell function is directly linked to the cell’s structure and synaptic connections. Retinal ganglion cell dendrite structure dynamically changes following injury, with reports of a temporary increase in dendritic complexity three weeks after optic nerve crush [182], and increased dendritic field size of surviving retinal ganglion cells following IOP elevation via episcleral vein cauterization in the rat [183]. However, the functional status of the retina was not measured in these studies, and further work is required to elucidate the structural changes that retinal ganglion cells undergo when exhibiting signs of recovery. A number of neurotrophic factors have demonstrated neuroprotective properties in models of glaucoma. Application of BDNF in a retinal explant model also markedly reduced dendritic degeneration, even if application was delayed following enucleation and explantation [184]. Co-administration of glial cell line-derived neurotrophic factor and ciliary neurotrophic factor not only demonstrated a synergistic neuroprotective effect on retinal ganglion cell survival, but also demonstrated evidence of stimulated intraretinal axon growth [185], supporting the potential for axonal plasticity. While therapeutics aimed at structural recovery and regeneration are unlikely to be in clinical use in the near future, evidence of functional recovery alone is much better documented.

### 4.2. Preclinical and Clinical Evidence of Functional Recovery

Signs of retinal ganglion cell functional recovery have been demonstrated in models of acute and chronic IOP elevation. Acute models of IOP elevation, in which IOP is increased to sub-ischemic levels via anterior chamber cannulation, have provided a highly reproducible model of acute IOP stress in animal models. Electroretinogram (ERG), a compound field potential generated by retinal cells in response to stimuli, continues to be an important tool to assess retinal ganglion cell function in animal models and humans. Pattern ERG (PERG), which specifically assesses retinal ganglion cell function (since it is abolished following optic nerve transection, in addition to end-stage neurodegeneration in DBA/2J mice and optic nerve crush [8,186,187,188]), is grossly proportional to the number of functioning or remaining retinal ganglion cells at earlier stages [189]. In contrast, other variations of the ERG contain responses from other inner retinal neurons within their waveforms, such as in the scotopic threshold response (STR) and photopic negative response (PhNR) [190]. Nevertheless, these maintain a robust method to assess retinal ganglion cell function and report similar ratios of change in glaucoma, and can be more appropriate than PERG in patients with refractive error or reduced optical clarity [191]. Selective retinal ganglion cell dysfunction, as measured using the STR in the ERG, occurs up to 50 mmHg [192]. Importantly, retinal ganglion cells demonstrated recovery in function following the normalization of IOP in young mice and rats [193,194]. However, the magnitude of acute IOP elevation affects the capacity to recover function. Increasing IOP to 70 mmHg for increasing durations led to progressively slower STR recovery [194], repeated IOP injury led to a cumulative effect on retinal ganglion cell dysfunction, and irreversible functional loss was observed four weeks after a sustained IOP insult (70 mmHg for 105 min in rats [195]). Chronic ocular hypertension models have also demonstrated functional recovery in pupil light reflex and ERG responses with up to eight weeks of ocular hypertension, but with irreversible retinal ganglion cell dysfunction following 12 weeks of ocular hypertension [179]. There is a clear age-related decline in potential for retinal ganglion cell functional recovery with older eyes (12–18 months) exhibiting reduced capacity to recover retinal function compared to younger eyes (3 months) following short-term acute IOP elevation in rodents [196,197]. While age increases susceptibility to acute IOP stress, this effect could be partially ameliorated via dietary restriction. Dietary restriction via intermittent fasting in 18-month-old mice improved inner retinal function recovery compared to controls [198]. These mice also showed reduced oxidative stress levels and significant improvement in mitochondrial oxidative phosphorylation enzyme activity. Axonal transport is impaired by acute IOP elevation, but returns rapidly following IOP normalization in rats and non-human primates [199,200], which may be a critical process for maintaining retinal ganglion cell viability.

Patients with early glaucoma have shown improvement in PERG following IOP lowering with topical treatment, whereas eyes with advanced disease showed little change [201]. We have also demonstrated the potential for short-term improvements in the PhNR following IOP lowering [202]. Three months after commencing a new IOP-lowering therapy, the treated group demonstrated significant improvement in the PhNR compared to baseline. Long-term, sustained improvement in visual field measurements have been shown after IOP reduction with trabeculectomy surgery [178]. Over five years post-surgery, 44% of visual field locations demonstrated improvement in the treated group, with 57% of operated eyes demonstrating ≥10 improving visual field locations. There has also been evidence of improved contrast sensitivity following glaucoma treatment. Sustained improvements to contrast sensitivity has been shown for up to three years post-trabeculectomy [177], where the change was directly correlated to a reduction in IOP. Temporary improvement to contrast sensitivity has been shown in people with glaucoma via topical and subconjunctival administration of glucose [203,204], and we have recently demonstrated improvement in retinal ganglion cell function in glaucoma following a 3-month course of nicotinamide [66]. It is worth noting that patients receiving nicotinamide continued to receive IOP-lowering therapeutics, suggesting an additive effect of supplementation with nicotinamide. These support the potential of further, metabolic-based interventions and continued research into nicotinamide as a viable long-term therapeutic. While many patients will be unresponsive to IOP lowering, changes to diet and exercise may yet yield some positive effects on visual decline, stabilization, or some level of recovery, even in isolation.

## 5. Conclusions and Clinical Perspectives

In conclusion, a number of diet- and exercise-mediated interventions demonstrate the potential for neuroprotective action in glaucoma. Further work in animal models will be necessary to understand the mechanisms of these protections, and a greater understanding of retinal ganglion cell metabolism will further enable this. The majority of clinical support for these diet and exercise interventions will require controlled clinical trials to better ascertain neuroprotective or functional benefit in glaucoma. The commercial availability of a number of these interventions suggests that they could be rapidly translated into clinical care with sufficient supportive data. However, a number of barriers still remain to conduct such studies. A significant impediment to developing new therapies for glaucoma includes the extended timeframe and budget required to demonstrate a treatment effect. The UKGTS could detect a difference in visual field in patients receiving glaucoma treatment versus placebo only after 12 months and a rigorous testing regime that is difficult to achieve in standard clinical care [205]. Development of clinical tools that are sensitive to changes in retinal structure and function over months instead of years will have a profound impact on shortening clinical trial durations and fast-tracking therapeutic development. There is a great need for additional therapies that can work synergistically with current IOP-lowering therapeutics. However, different types of glaucoma may require different additive therapies, leading to a more personalized therapeutic approach to glaucoma treatment that takes the general health of patients into the equation. With this in mind, patients seeking to explore the interventions presented here should always consult their glaucoma specialist in order to determine what is right for them.

## Data Availability

Not applicable.
